# Selective protection of the cerebellum against intracerebroventricular LPS is mediated by local melatonin synthesis

**DOI:** 10.1007/s00429-013-0686-4

**Published:** 2013-12-22

**Authors:** Luciana Pinato, Sanseray da Silveira Cruz-Machado, Daiane G. Franco, Leila M. G. Campos, Erika Cecon, Pedro A. C. M. Fernandes, Jackson C. Bittencourt, Regina P. Markus

**Affiliations:** 1 Laboratory of Chronopharmacology, Department of Physiology, Institute of Biosciences, University of São Paulo (USP), São Paulo, SP 05508-090 Brazil; 2 Department of Speech, Language and Hearing Therapy, São Paulo State University (UNESP), Marilia, SP 17525-900 Brazil; 3 Department of Anatomy, Institute of Biomedical Sciences, University of São Paulo (USP), São Paulo, SP 05508-900 Brazil

**Keywords:** Pineal gland, Arylalkylamine *N*-acetyltransferase (AA-NAT), Immune-pineal axis, Neuroinflammation, Melatonin receptors, Nuclear factor kappa B (NF-κB)

## Abstract

Although melatonin is mainly produced by the pineal gland, an increasing number of extra-pineal sites of melatonin synthesis have been described. We previously demonstrated the existence of bidirectional communication between the pineal gland and the immune system that drives a switch in melatonin production from the pineal gland to peripheral organs during the mounting of an innate immune response. In the present study, we show that acute neuroinflammation induced by lipopolysaccharide (LPS) injected directly into the lateral ventricles of adult rats reduces the nocturnal peak of melatonin in the plasma and induces its synthesis in the cerebellum, though not in the cortex or hippocampus. This increase in cerebellar melatonin content requires the activation of nuclear factor kappa B (NF-κB), which positively regulates the expression of the key enzyme for melatonin synthesis, arylalkylamine *N*-acetyltransferase (AA-NAT). Interestingly, LPS treatment led to neuronal death in the hippocampus and cortex, but not in the cerebellum. This privileged protection of cerebellar cells was abrogated when G-protein-coupled melatonin receptors were blocked by the melatonin antagonist luzindole, suggesting that the local production of melatonin protects cerebellar neurons from LPS toxicity. This is the first demonstration of a switch between pineal and extra-pineal melatonin production in the central nervous system following a neuroinflammatory response. These results have direct implications concerning the differential susceptibility of specific brain areas to neuronal death.

**Electronic supplementary material:**

The online version of this article (doi:10.1007/s00429-013-0686-4) contains supplementary material, which is available to authorized users.

## Introduction

The innate immune response is a stereotyped process that enables an organism to overcome adverse conditions. The pro-inflammatory phase of the innate immune response is followed by an adaptive reparative phase in which the production of anti-inflammatory agents balances potentially harmful substances. The resolution phase restores homeostatic conditions and tissue function (McCall et al. 2011). In the central nervous system (CNS), the pro-inflammatory phase must be highly regulated to prevent conditions that favor neuronal death and disease (Schmidt et al. 2005; Sastre et al. 2006; Qian et al. 2010; Tansey and Goldberg 2010).

Melatonin, the product of the acetylation and methylation of serotonin, is synthesized by the pineal gland in response to signals from the central biological clock, which is, in turn, regulated by the environmental light–dark cycle. This hormone translates the dark phase to the organism, synchronizing circadian (Hardeland et al. 2012) and seasonal rhythms (Barrett and Bolborea 2012). Melatonin also plays a role in the defense of the organisms (Tan et al. 2010a). Extra-pineal melatonin synthesis occurs in several organs, including the retina (Gern and Ralph 1979; Tosini and Menaker 1996), the gastrointestinal tract (Raikhlin et al. 1975; Bubenik 2001), immune competent cells (Finocchiaro et al. 1991; Carrillo-Vico et al. 2004; Martins et al. 2004; Pontes et al. 2006) and astrocytes in culture (Liu et al. 2007b). The paracrine action of extra-pineal melatonin is important for the organization of immune responses (Carrillo-Vico et al. 2013).

Recently, the molecular basis for the bidirectional communication between the immune system and the pineal gland was extensively reviewed (Markus and Ferreira 2011; Markus et al. 2013). The activation of the transcription factor nuclear factor kappa B (NF-κB) results in specific effects that depend on the cellular milieu. In the pineal gland, NF-κB activation suppresses the expression of the enzyme arylalkylamine N-acetyltransferase (AA-NAT), which converts serotonin to the melatonin precursor N-acetylserotonin (NAS) (Fernandes et al. 2006; Markus et al. 2007; Cecon et al. 2010; Da Silveira et al. 2010; Carvalho-Sousa et al. 2011; Fernandes and Markus 2011; Da Silveira et al. 2012). Conversely, in bone marrow monocytes, colostrum defense cells, macrophages and lymphocytes, NF-κB activation induces the synthesis of melatonin (Conti et al. 2000; Carrillo-Vico et al. 2004; Pontes et al. 2006; Muxel et al. 2012). These differential effects depend on the cell environment and the subtype of dimer that is translocated to the nucleus (Muxel et al. 2012; Pires-Lapa et al. 2013). Because the nuclear translocation of NF-κB is triggered by toll-like receptors (TLRs) or receptors for pro-inflammatory cytokines, in both pinealocytes and immune competent cells, this transcriptional pathway is considered the basis for the switch of melatonin production from the pineal gland to immune competent cells (Markus et al.^ 2007^, ^2013^).

Understanding the regulation and dynamics of melatonin synthesis in physiological and pathophysiological conditions is essential because of the wide range of beneficial actions of melatonin, especially those related to neuroprotection (Wang 2009; Cardinali et al. 2010; Cecon and Markus 2011; Srinivasan et al. 2011). For instance, dampened melatonin rhythms have been described in several neurological disorders, including Alzheimer’s disease, autism, schizophrenia and multiple sclerosis (Hardeland 2012). This dampening suggests pineal dysfunction, most likely related to neuroinflammation, which might favor disease development.

Although astrocytes and microglia were previously demonstrated to express both enzymes required for melatonin biosynthesis (Uz et al. 2002; Jimenez-Jorge et al. 2007; Liu et al. 2007b; Tan et al. 2010b), no information is available regarding the neuroprotection of specific brain areas due to local synthesis of melatonin. In the current study, we investigated whether neuroinflammation induces a shift in melatonin synthesis from the pineal gland to other brain areas. We also studied the putative functions of locally produced melatonin. Specifically, we tested whether the Immune-Pineal Axis theory can be applied in the brain and whether it confers special protection to areas that can locally produce melatonin following injury. Our findings reveal that the acute neuroinflammatory response induced by intracerebroventricular (icv) injection of lipopolysaccharide (LPS) decreases nocturnal levels of circulating melatonin, while inducing its local production in specific brain areas. Moreover, local melatonin synthesis correlated with lower neuronal death, which offers new insight into why some brain areas are more susceptible to neurodegeneration than others.

## Material and methods

### Animals

Male Wistar rats (2 months old, 180–200 g) were obtained from the animal facility of the Department of Physiology (IB-USP, São Paulo, Brazil). They were subjected to a 12:12 h light/dark cycle (lights on at 07:00 hours = Zeitgeber time zero or ZT 0) and received water and food ad libitum. The animals were killed by decapitation or transcardial perfusion with a fixative solution. Procedures were approved by the IB-USP Ethical Committee (license number 082/2008) and carried out in compliance with the recommendations of the National Council on Experimental Animal Control (CONCEA).

### Drugs and reagents

Lipopolysaccharide (LPS, from *E. coli* serotype 0127:B8), glycerol buffer, pyrrolidinedithiocarbamate (PDTC), luzindole, trypsin, trypsin inhibitor, melatonin, 4-(2-hydroxyethyl)-1-piperazineethanesulfonic acid (HEPES), DPX, 3-(4,5-dimethylthiazol-2-yl)-2,5-diphenyltetrazolium bromide (MTT) and the monoclonal anti-GFAP Cy3-conjugated (S9205), rabbit anti-AA-NAT (S0939) and anti-rabbit FITC-conjugated IgG (F7512) antibodies were obtained from Sigma-Aledrich Chemical (St. Louis, MO, USA). Ascorbic acid and ethylenediaminetetraacetic acid (EDTA) were purchased from Merck (Rio de Janeiro, RJ, Brazil). 6-Diamidino-2-phenylindole (DAPI), was obtained from Life Technologies (Carlsbad, CA, USA). The mouse anti-NeuN monoclonal antibody was obtained from Millipore Co. (Billerica, MA, USA), the mouse anti-ED1 (ab31630) and goat anti-IBA1 (ab5076) antibodies were purchased from Abcam (Cambridge, UK), and the donkey anti-mouse Cy3-conjugated IgG (715165150) antibody was purchased from Jackson ImmunoResearch (West Grove, PA, USA). Fluoro-Jade B was obtained from Chemicon International Inc. (Temecula, CA, USA). Dulbecco’s modified Eagle’s medium (DMEM), fetal calf serum and penicillin/streptomycin were obtained from GIBCO BRL Products (Grand Island, NY, USA).

### Surgical procedure

Rats were anesthetized using ketamine (100 mg/kg) and xylazine (40 mg/kg, i.p.). A guide cannula (Plastics One, Roanoke, VA, USA) was lowered via a stereotaxic arm to a depth of 3.5 mm from the dura mater into the right lateral ventricle (1.4 mm lateral and 0.4 mm posterior to the bregma), permanently affixed to the skull using anchor screws (Plastics One, Roanoke, VA, USA) and dental acrylic (Jet Clássico, SP, Brazil) and covered with a dummy cannula (Plastics One, Roanoke, VA, USA). After surgery, the animals were placed in a warm chamber to recover from anesthesia before being placed back in their home cages and maintained for 7–10 days prior to experimental procedures.

A total of 5 μL (1 μL/min) of LPS (3 μg/injection) or saline was injected in the lateral ventricle through an infusion probe (Plastics One, Roanoke, VA, USA) connected to a Hamilton gastight syringe (Hamilton Co., Reno, NV, USA) at ZT 6. After 12 h (at ZT 18), the animals were killed by decapitation, and plasma or brain tissue samples were collected to determine melatonin concentration and identify serotonin derivatives. For immunohistochemical analysis, the animals were anesthetized and transcardially perfused (saline followed by 4 % paraformaldehyde) at ZT 18. Inhibition of NF-κB activity and melatonin receptors was achieved by injecting PDTC (100 μg/kg, i.p., at ZT 4) and luzindole (10 μM, icv, at ZT 5 and ZT 12), respectively.

Pinealectomy was performed in rats anesthetized, as described above, and placed in a stereotaxic apparatus. Through an incision in the mid line of the skull, the skin and muscles were pushed, the skull exposed and the bones were drilled with a micromotor and a dental bur. A circular piece of skull with approximately 4 mm of diameter made in the lambda point allowed the visualization of the sagittal and the transverse sinuses. The pineal gland, which is located just below the confluence of the venous, was removed. In the sham-operated animals, the same procedure was performed except pineal extraction. The returning of the disc-shaped bone closed the skull, and the scalp was sutured with suture clips. The effectiveness of the pinealectomy was evaluated by macroscopic post-mortem examination and by determining the melatonin level in the plasma at nighttime (Supplementary Fig. 1).

### Tissue collection

For immunohistochemical analysis, animals were transcardially perfused with 150 mL saline solution followed by 400 mL of a cold (±4 °C) 4 % formaldehyde fixative solution at pH 9.5. The brains and pineal glands were removed and post-fixed in the same fixative solution with 30 % sucrose for 4 h at 4 °C. After fixation, the brains and pineal glands were cryoprotected with 30 % sucrose in 0.1 M phosphate buffer solution at pH 7.4 (PBS) and frozen. Before freezing, the pineal glands were embedded in Tissue Tek (Sakura, Torrance, CA, USA). The frozen blocks were cut in serial sections (20 μm thick) on a cryostat (Leica Microsystems Inc., Bannockburn, IL, USA) and collected in PBS. Serial coronal brain sections (30 μm thick) were cut with a cryomicrotome (Leica Microsystems Inc., Bannockburn, IL, USA) and collected in antifreeze solution (six series), one series per time was used for immunohistochemistry or Fluoro-Jade B staining.

### Immunofluorescence

The expression patterns of AA-NAT in the pineal glands and AA-NAT co-labeled with GFAP, NeuN, ED-1 or IBA1 were revealed using the indirect immunofluorescence method on free-floating sections.

First, the sections were incubated in blocking solution [1 % normal goat serum (NGS, Vector, Burlingame, CA, USA), 0.3 % Triton X-100 in 0.1 M PBS, pH 7.4] for 1 h at room temperature, followed by incubation with the primary antibodies diluted in the same solution (48 h at 4 °C). Next, the sections were rinsed with PBS and incubated with secondary antibodies diluted in a similar solution for 90 min. Nuclei were stained with DAPI. After rinsing, the brain sections were mounted on gelatin-coated slides and covered with a cover glass, using glycerol buffer as a mounting medium.

For double immunofluorescence, mouse monoclonal anti-GFAP Cy3-conjugated (1:2,000), mouse anti-NeuN (1:1,000), rabbit anti-AA-NAT (1:500), mouse anti-ED-1 (1:100) and goat anti-IBA1 (1:200) primary antibodies were used. The concentrations of the antibodies were chosen according to a concentration–response curve for each antibody. Anti-rabbit FITC-conjugated IgG (1:200) and donkey anti-mouse Cy3-conjugated IgG (1:200) secondary antibodies were used.

All procedures were repeated at least three times. Controls were performed by omitting the primary antibodies from the procedure and substituting normal serum from the same species. Staining was completely abolished under these conditions.

### Fluoro-Jade B staining

Cell death was detected by the Fluoro-Jade B method. The sections mounted on 2 % gelatin-coated slides were air-dried for at least 12 h. The slides were immersed in 1 % sodium hydroxide in 80 % alcohol for 5 min followed by 2 min in 70 % alcohol and 2 min in distilled water. The slides were then transferred to a solution of 0.06 % potassium permanganate for 10 min, rinsed and immersed in a staining solution prepared from a 0.01 % stock solution of Fluoro-Jade B and added to 0.1 % acetic acid vehicle solution at a final dye concentration of 0.0004 %. After 20 min in the staining solution, the slides were rinsed, placed on a slide warmer, cleared by immersion in xylene and coverslipped with DPX. Nuclei of undamaged cells were stained with DAPI and analyzed by epifluorescent microscopy.

### Cerebellar granule cell culture

Animals (7–8 days old) were decapitated, and each cerebellum was isolated, washed, cut into small pieces and incubated in 0.05 % trypsin dissolved in physiological solution (120 mM NaCl, 5 mM KCl, 1.2 mM KH_2_PO_4_, 1.2 mM MgSO_4_·7H_2_O, 25 mM NaHCO_3_ and 13 mM glucose, pH 7.4) for 40 min at 37 °C. Afterward, a trypsin inhibitor (0.06 %) was added to the solution, and the cells were isolated by mechanical dispersion followed by centrifugation (300*g*, 5 min). Cells were seeded (5 × 10^5^–10^7^ cells/well, 13.4 g/L DMEM, 10 % SFB, 25 mM K^+^, 44 mM NaHCO_3_, 50 U/mL penicillin and 50 μg/mL streptomycin) and maintained at 37 °C in a 5 % CO_2_ atmosphere for 7 days. The culture medium was replaced every 48 h. On day 7, the cell cultures were incubated with LPS (30 and 100 ng/mL) or vehicle for 24 h. The medium was collected to determine the melatonin content through an ELISA assay.

### Cell viability assay—MTT method

Cell viability was assessed in cerebellar cell cultures challenged with LPS (30 ng/mL, 24 h) in the presence or absence of luzindole (100 nM, 24 h). MTT was added to the wells at a final concentration of 5 mg/mL for 2 h. The reduced crystals of MTT (formazan) were dissolved in DMSO (30 min) and read at 540 nm (Molecular Devices SpectraMax 250). Viability was expressed as the percentage of the absorbance measured in control cells.

### Indoleamine determination

Levels of serotonin, NAS and 5-hydroxy-indoleacetic acid (5-HIAA) in the pineal gland were determined by high-performance liquid chromatography (HPLC) through electrochemical detection according to a previously described methodology (Da Silveira et al. 2010). Briefly, the glands were homogenized in ice-cold 0.1 M perchloric acid (120 μL) containing 0.02 % EDTA and 0.02 % sodium bisulfite. After centrifugation, 20 μL of the resulting supernatant was injected into the chromatographic system (Waters, Milford, MA, USA), which was isocratically operated. The mobile phase for serotonin, 5-HIAA and NAS (0.1 M sodium acetate, 0.1 M citric acid, 0.15 mM EDTA and 10 % methanol, pH 3.7) flowed at a rate of 0.95 mL/min through a 5-mm Resolve C 18 reversed-phase column (Waters, Milford, MA, USA). The detector potential was adjusted to  + 0.90 V versus an Ag/AgCl reference electrode.

The brain tissues were homogenized in ice-cold 25 mM Tris–Hcl pH 7.4 (400 μL) containing 1 mM EDTA and 1 mM EGTA. Melatonin concentration in the plasma, tissues and culture media was determined using an ELISA method according to the manufacturer’s specifications (rat melatonin ELISA kit, IBL, Germany).

### Imaging and data analysis

Double-labeled sections were analyzed and photographed using a confocal laser-scanning microscope (LSM 510, Zeiss, Baden-Wurttemberg, Germany). HeNe 543/633, argon (excitation 488 nm) and enterprise (excitation 364 nm) lasers were used for Cy3 (560 nm emission), FITC (505 nm emission) and DAPI (435–485 nm emission) imaging, respectively. To assess possible co-localization, images in the red and green channels were captured. Co-localization appeared yellow in the merged images of confocal optical sections. The Fluoro-Jade B staining was analyzed with a Nikon E-1000 epifluorescence photomicroscope (Melville, NY, USA) with a cool snap camera (Pro-color, Media Cybernetics, Silver Spring, MA, USA). The Fluoro-Jade B positive cells were counted in all the sections of each series using Image-Pro Plus software (Media Cybernetics Inc., Silver Spring, MD, USA).

The relative contents of AA-NAT and other cell markers were quantified by measuring the optical density (OD) of fluorescence intensities. For each experimental series, all high-resolution z-stack images were acquired with identical settings. The relative intensities were measured from the raw images using Image-Pro Plus software (Media Cybernetics Inc., Silver Spring, MD, USA) under fixed thresholds across all slides.

### Statistical analysis

The data are presented as the mean ± standard error of the mean. Differences between two or more groups were tested using Student’s *t* test or ANOVA followed by the Newman–Keuls test, respectively. Values of *p* < 0.05 were considered statistically significant.

## Results

### LPS (icv) decreases melatonin synthesis in the pineal gland

**Fig. 1:**
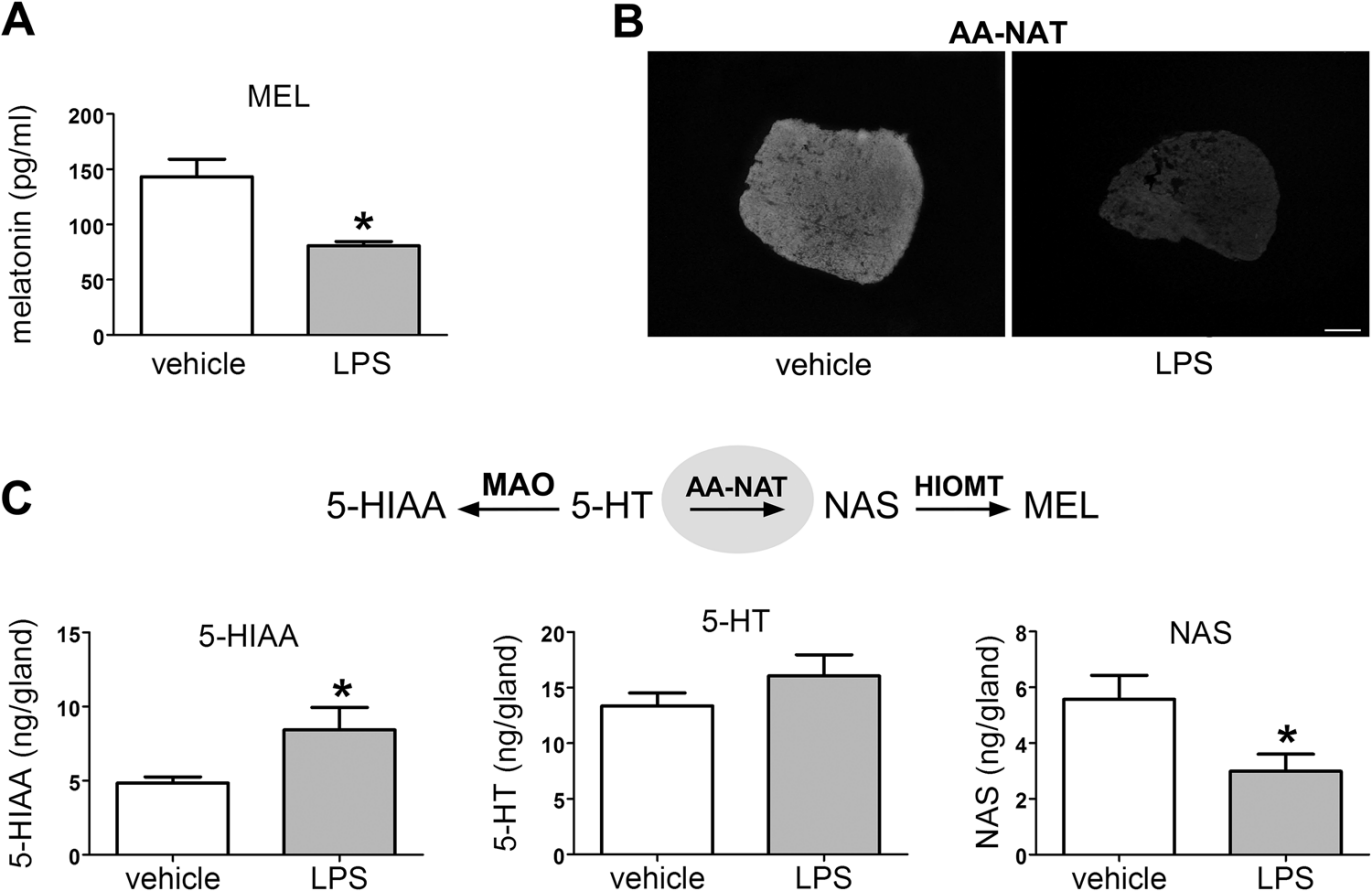
LPS (icv) and melatonin production by the pineal gland. **a** Plasma melatonin levels 12 h after the injection of vehicle (*n* = 8) or LPS (3 μg, ZT 6, icv, *n* = 9). **b** Representative photomicrographs of AA-NAT immunoreactivity in pineal sections from vehicle- and LPS-treated rats. **c** 5-Hydroxy-indoleacetic acid (5-HIAA), serotonin (5-HT) and N-acetylserotonin (NAS) contents in the pineal glands of vehicle- and LPS-treated rats (*n* = 6). The data are expressed as the mean ± SEM with *p < 0.05. *Scale bar* 500 μm

LPS (3 μg, icv, ZT6) significantly reduced the nocturnal concentration of melatonin in the blood as well as the immunoreactivity for AA-NAT in pineal sections from rats killed 12 h later (ZT 18) (Fig. 1a, b). The content of the melatonin precursor NAS, the first product generated by AA-NAT activity, was also reduced. The level of 5-HIAA was significantly increased while serotonin content was not affected (Fig. 1c). These data suggest that a reduction in the availability of AA-NAT, which converts serotonin into NAS, impairs the pineal synthesis of melatonin, diverting serotonin to the monoaminooxidase (MAO) route. This change in pineal function translates into a reduction in the nocturnal surge of melatonin in the plasma.

### LPS alters AA-NAT expression in specific brain areas

Given that LPS induces the transcription of the *Aanat* gene in macrophages through the activation of NF-κB (Muxel et al. 2012) we evaluated whether the same inflammatory agent also alters the expression of this enzyme in different areas and cell types (neurons and glial cells) of the central nervous system.

**Fig. 2:**
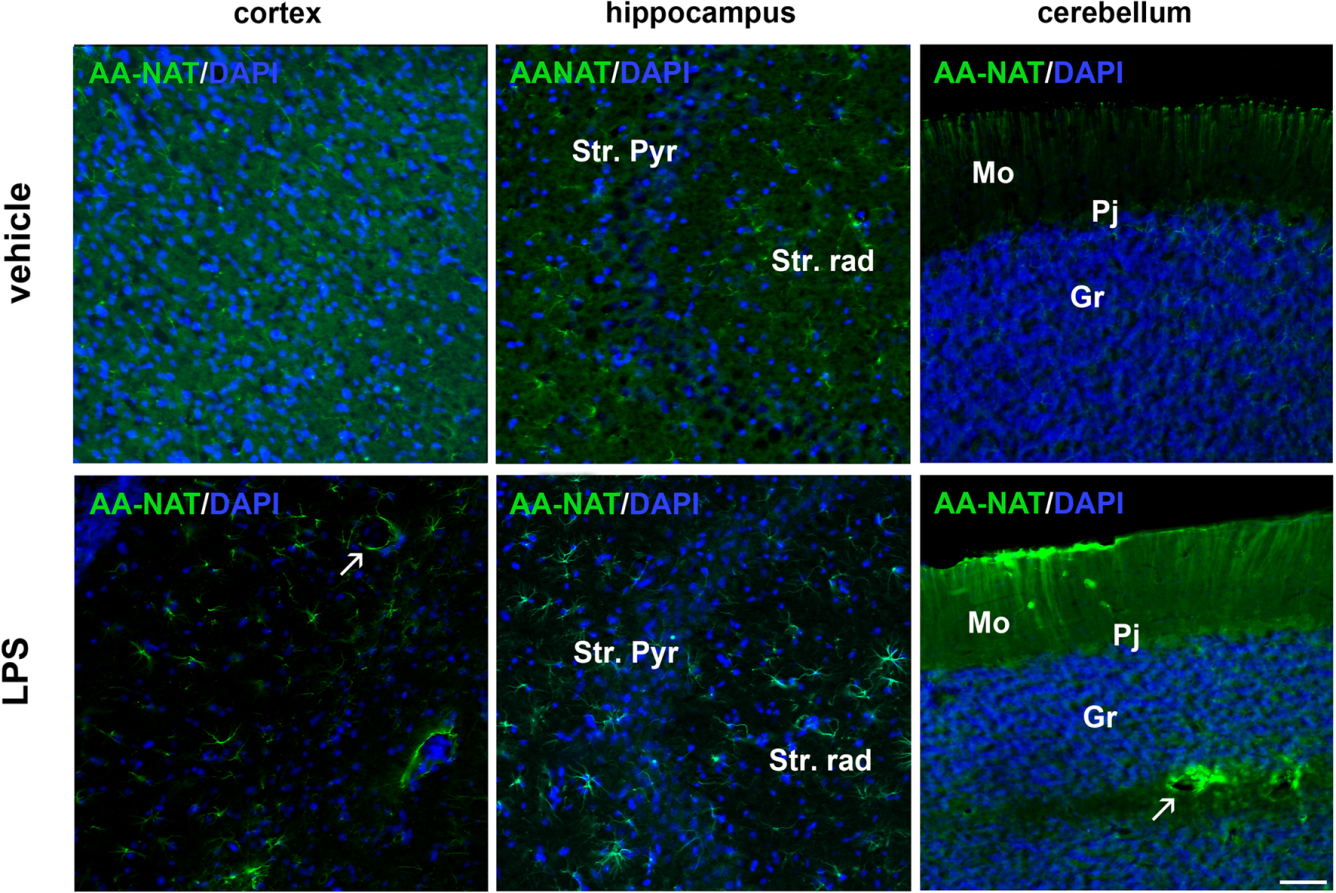
LPS increased AA-NAT expression. **a** Representative confocal photomicrographs of immunolabeled rat coronal sections of the cortex, the hippocampus [containing the pyramidal cell layer (Str. Pyr) and the stratum radiatum (Str. rad)] and the cerebellum [containing the molecular (Mo), granular (Gr) and Purkinje cell (Pj) layers]. Qualitative analysis of AA-NAT (*green*) expression in the vehicle and LPS groups. The cell nuclei were stained with DAPI (*blue*). *Arrows* indicate cells surrounding blood vessels. *Scale bar* 100 μm

A general view of AA-NAT expression in the cortex, hippocampus and cerebellum of rats treated with vehicle or LPS suggests that LPS treatment leads to an increase in AA-NAT staining in the cerebellum (Fig. 2). In the hippocampus and cortex, AA-NAT-immunoreactive (ir) cells changed in form after LPS treatment. In tissues from treated animals astrocytes present star-shaped forms while in control tissues the fluorescence was observed as dots and the cells had no specific morphology, suggesting that the cellular localization of AA-NAT-ir is modified by LPS (Fig. 2). In the cerebellum of LPS-treated rats AA-NAT-ir increased in the molecular, granular and Purkinje cell layers (Fig. 2).

**Fig. 3:**
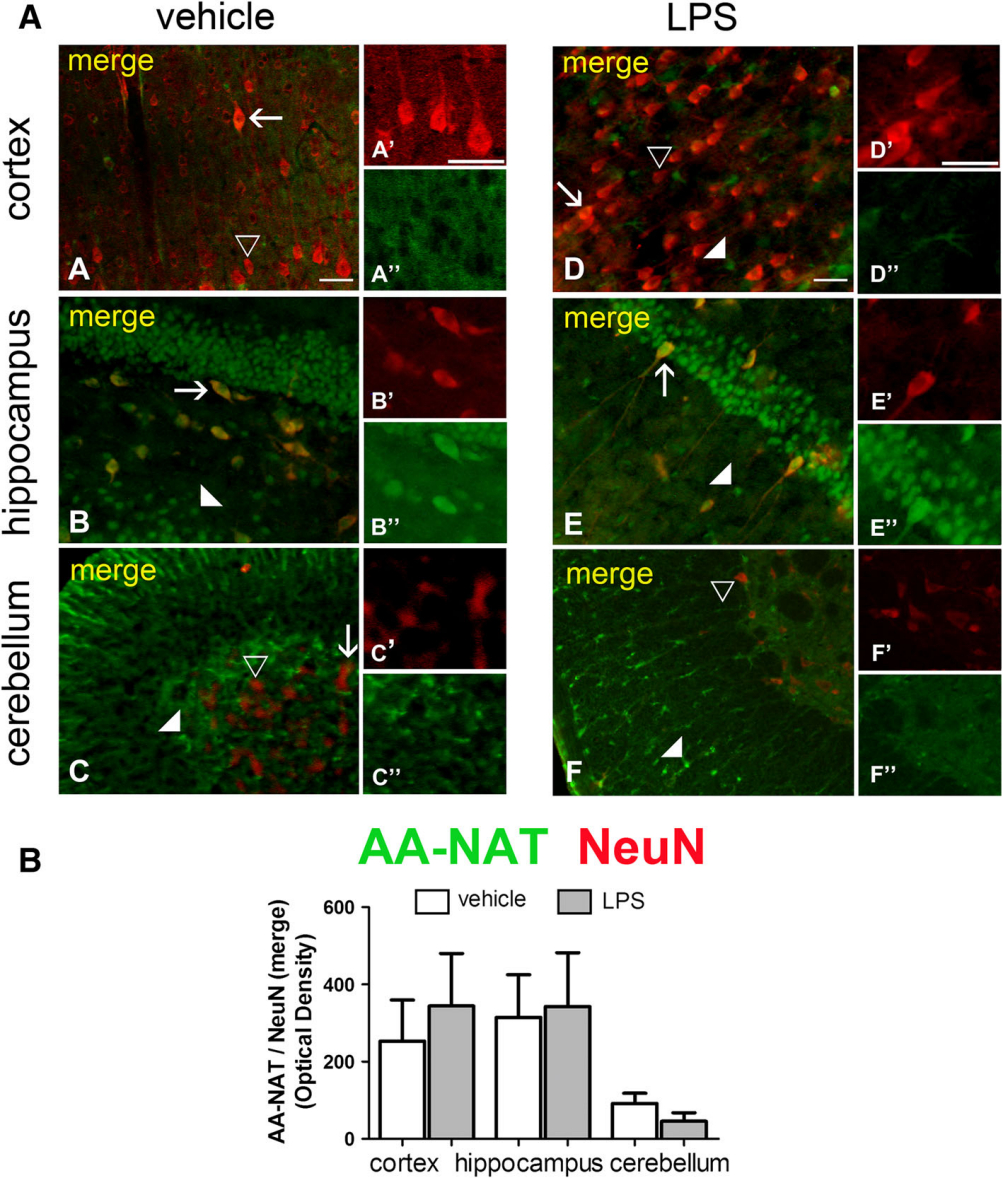
LPS did not increase AA-NAT expression in NeuN-immunoreactive neurons. **a** Representative confocal photomicrographs of double-immunolabeled coronal sections of the cortex, hippocampus and cerebellum of vehicle- and LPS-treated animals, showing immunoreactivity to AA-NAT (*green*) and NeuN (*red*). AA-NAT-ir cells (*white arrowhead*), NeuN-ir cells (*black arrowhead*) and the co-localization of AA-NAT and NeuN (*yellow* in merged channels, *arrow*) in the vehicle and LPS groups. *Scale bar* 100 μm. **b** Quantification of immunohistochemical results showing no increase in the optical density of AA-NAT and NeuN-ir co-localization in the three analyzed areas (*n* = 4 animals per group). *Significantly different (*p* < 0.05)

To further determine which cells were expressing AA-NAT, we performed double-staining for AA-NAT with NeuN, GFAP, ED-1 and IBA1, which are markers for neurons, astrocytes and two different stages of activated microglia, respectively. In cortical and cerebellar sections, AA-NAT and NeuN staining did not co-localize in treated or untreated animals (Fig. 3). However, in the hippocampus, some NeuN-positive cells co-expressed AA-NAT (Fig. 3a). Interestingly, cerebellar Purkinje cells, which are known to be non-reactive to NeuN (Mullen et al. 1992; Wolf et al. 1996; Weyer and Schilling 2003), but can be identified by their morphology, exhibited AA-NAT-ir. No significant difference in the co-localization of AA-NAT-ir with NeuN-ir was detected between vehicle- and LPS-treated rats (Fig. 3b).

**Fig. 4:**
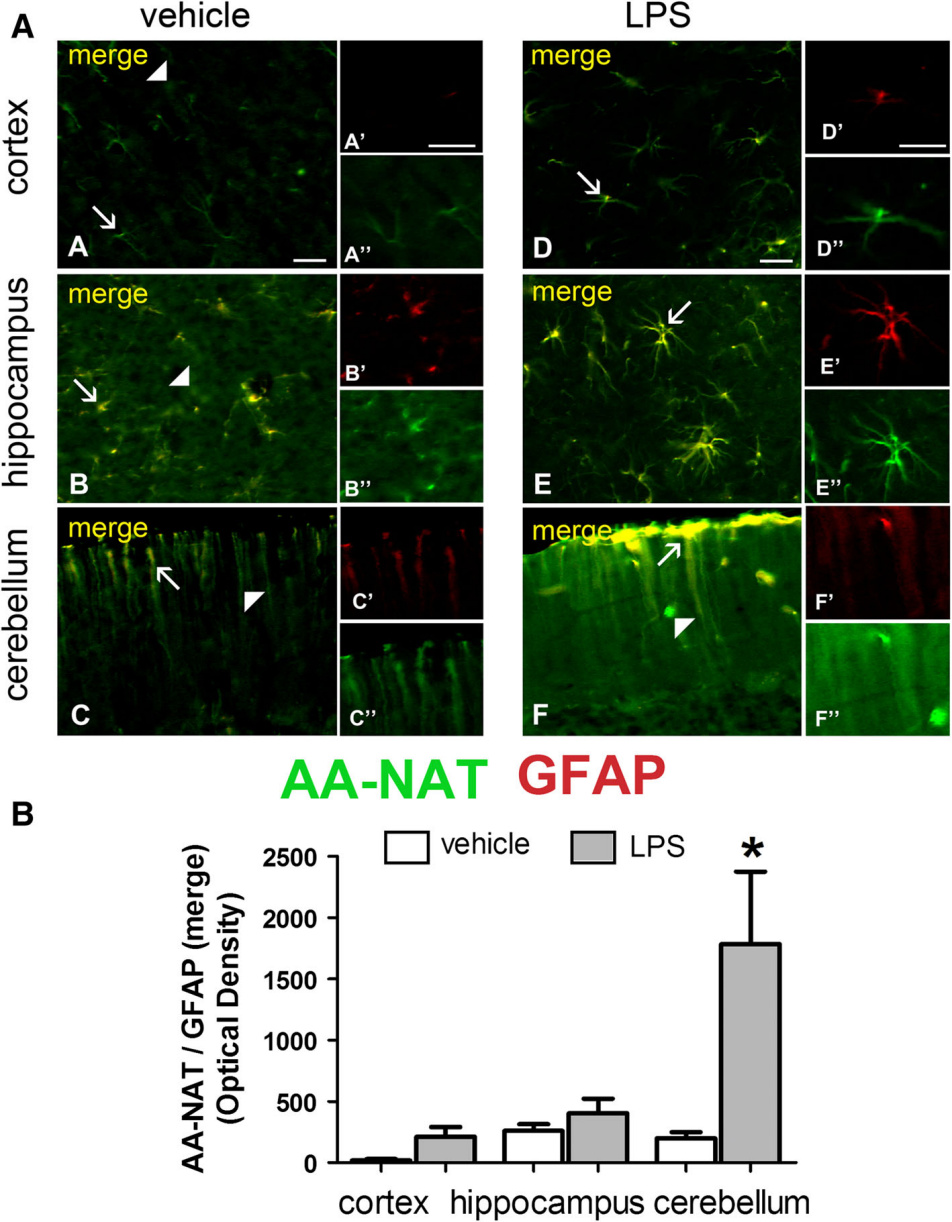
LPS increased AA-NAT expression in GFAP immunoreactive astrocytes. **a** Representative confocal photomicrographs of double-immunolabeled coronal sections of the cortex, hippocampus and cerebellum of vehicle- and LPS-treated animals, showing the immunoreactivity to AA-NAT (*green*) and GFAP (*red*). AA-NAT-ir cells (*white arrowhead*), GFAP-ir cells (*black arrowhead*) and the co-localization of AA-NAT and GFAP (see merged image, *arrow*) in the vehicle and LPS groups. *Scale bar* 100 μm. **b** Quantification of immunohistochemical results showing increased optical density of AA-NAT and GFAP-ir co-localization in the cerebellum (*n* = 4 animals per group). *Significantly different (*p* < 0.05)

The results were quite different for glial cells. In the cortex and hippocampus, LPS induced clear morphological changes in astrocytes, which exhibited notable branch elongation (Fig. 4a). In addition, AA-NAT-ir co-localized with GFAP in both vehicle- and LPS-treated groups in all analyzed brain areas. In the cerebellum, astrocytes were positively stained for both GFAP and AA-NAT throughout the molecular layer. LPS induced a significant increase in the co-localization of AA-NAT-ir with GFAP-ir only in the cerebellum (Fig. 4b). Although the image in Fig. 4b suggests much higher levels of AA-NAT-ir in cortical GFAP-ir cells, Student’s *t* testing revealed a probability of 8 %.

**Fig. 5:**
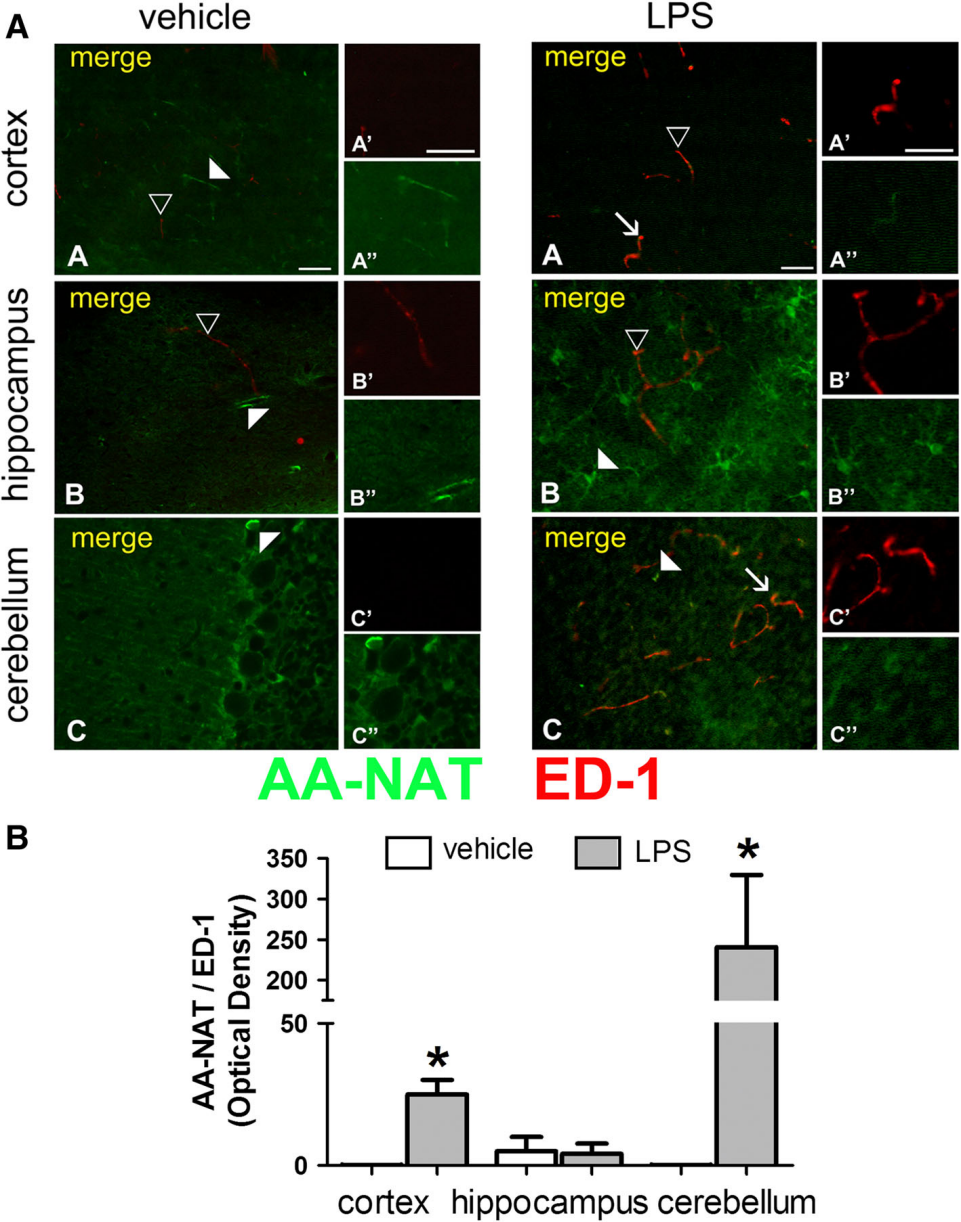
LPS induced AA-NAT expression in ED-1 immunoreactive cells. **a** Representative confocal photomicrographs of double-immunolabeled coronal sections of the cortex, hippocampus and cerebellum of vehicle- and LPS-treated animals, showing the immunoreactivity to AA-NAT (*green*) and ED1 (*red*). Representative images of AA-NAT-ir cells (*white arrowhead*), ED1-ir cells (*black arrowhead*) and the co-localization of AA-NAT and ED1 (*arrow*) in the LPS group. *Scale bar* 100 μm. **b** Quantification of immunohistochemical results, showing that after LPS treatment, AA-NAT co-localized with ED1 in a small number of cells in the cortex, but more prominently in the cerebellum (*n* = 4 animals per group). *Significant different (*p* < 0.05)

**Fig. 6:**
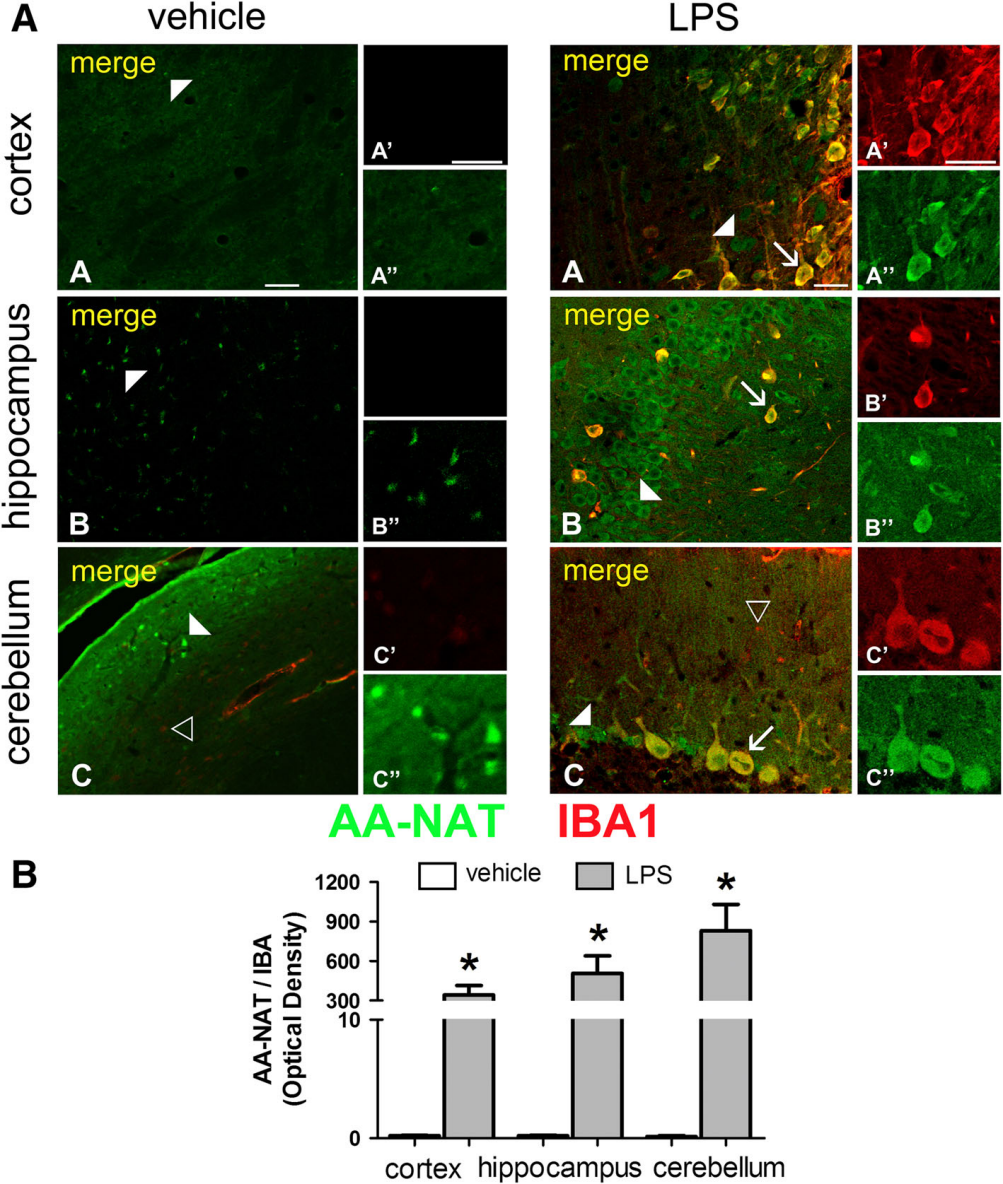
LPS induced AA-NAT expression in IBA-1immunoreactive cells. **a** Representative confocal photomicrographs of double-immunolabeled coronal sections of the cortex, hippocampus and cerebellum of vehicle- and LPS-treated animals, showing the immunoreactivity to AA-NAT (*green*) and IBA1 (*red*). Representative images of AA-NAT-ir cells (*white arrowhead*), IBA1-ir cells (*black arrowhead*) and the co-localization of AA-NAT and IBA (*arrow*) in the LPS group. *Scale bar* 100 μm. **b** Quantification of immunohistochemical results showing clear increase in AA-NAT and IBA1 co-localization in all three brain areas in the LPS group (*n* = 4 animals per group). *Significantly different (*p* < 0.05)

Two different stages of activation were detected in microglia cells, and the expression of AA-NAT-ir varied according to the brain area studied. Immunoreactivity to both ED-1 (Fig. 5) and IBA1 (Fig. 6) was significantly higher in brain slices from LPS-treated animals. LPS induced significant increase in the co-localization of AA-NAT-ir with ED-1 in the cortex and the cerebellum (Fig. 5b). IBA1-positive microglia cells, which are round in shape, co-labeled with AA-NAT after LPS treatment in all analyzed areas (Fig. 6a). The marked increase in AA-NAT and IBA1 double-staining following LPS treatment in the cortex, hippocampus and cerebellum was evident by quantitative analysis (Fig. 6b).

### LPS induces melatonin synthesis in the cerebellum but not in the cortex or the hippocampus

To evaluate the functionality of AA-NAT expression in the cortex, hippocampus and cerebellum, we measured the amount of melatonin in vehicle- and LPS-treated animals in the three areas. In addition, a group of animals was pinealectomized prior to vehicle or LPS icv injection to distinguish between melatonin originating from the plasma (i.e., pineal-derived melatonin) and locally produced melatonin.

**Fig. 7:**
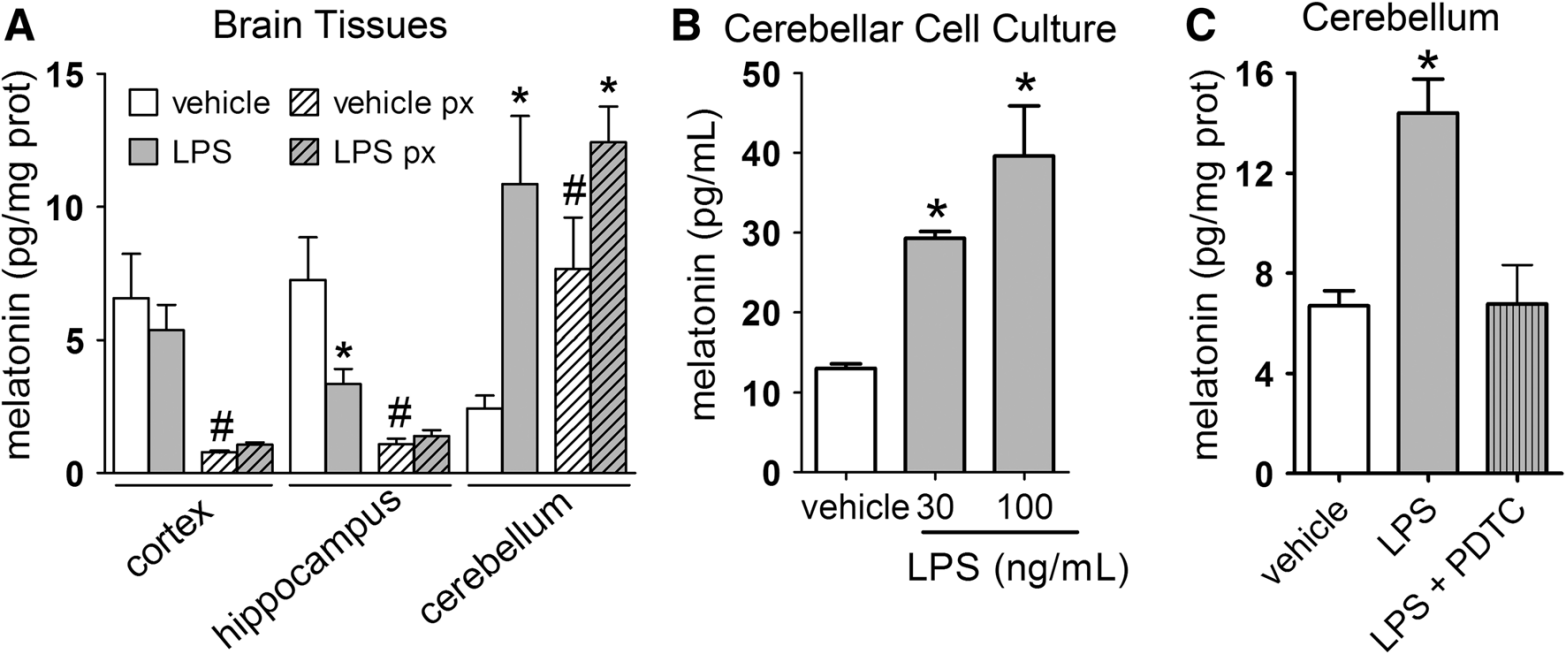
LPS-induced changes in melatonin content vary according to the brain structures. **a** Melatonin levels in the cortex, hippocampus and cerebellum of vehicle- and LPS-treated animals submitted or not to pinealectomy (px).*LPS ≠ vehicle, ^#^pinealectomized (px) ≠ control, *n* = 9; *p* < 0.05. **b** Melatonin content in cultured cerebellar cells challenged with LPS (30 and 100 ng/mL) for 12 h. *Significantly different from vehicle *p* < 0.05; *n* = 4. **c** Melatonin levels in the cerebellum of vehicle- and LPS-(3 μg/5 μL, icv, ZT 6; *gray bar*) treated animals in the presence or absence of the blockage of proteasome with PDTC (100 μg/kg, i.p., ZT 4) 2 h before LPS administration (*gray hachured bar*). The data are expressed as the mean ± SEM. Significantly different *p < 0.05; *n* = 6–9

LPS treatment had no effect on melatonin levels in the cortex, but produced a significant reduction in the hippocampus. Conversely, a fourfold increase was detected in the cerebellum of LPS-treated animals (Fig. 7a). In the pinealectomized groups, melatonin content was significantly reduced in the cortex and hippocampus of both vehicle- and LPS-treated animals. In contrast, the LPS-induced increase in melatonin production observed in the cerebellum was not reversed by pinealectomy. In addition, pinealectomy alone induced an increase in melatonin content specifically in the cerebellum, which was further enhanced after LPS treatment, confirming that cerebellar cells synthesize melatonin (Fig. 7a). These observations were further confirmed by in vitro experiments in which LPS (30 or 100 ng/mL for 24 h) induced melatonin production in cultured cerebellar cells in a dose-dependent manner (Fig. 7b).

It has previously been shown that AA-NAT gene expression and thus melatonin synthesis in RAW246.7 macrophages are driven by the transcription factor NF-κB (Muxel et al. 2012), which is classically activated following LPS stimulus. Therefore, we evaluated whether NF-κB also mediates melatonin synthesis in the cerebellum. Indeed, inhibition of NF-κB binding to DNA by injection of PDTC 2 h prior to LPS injection reversed the induction of melatonin synthesis in the cerebellum (Fig. 7c).

In summary, LPS differentially affected cerebellar glial cells relative to the hippocampal and cortical cells. The significant increase in AA-NAT-ir results in the synthesis of melatonin by the cerebellum.

### Local LPS-induced melatonin synthesis protects cerebellar neurons

**Fig. 8:**
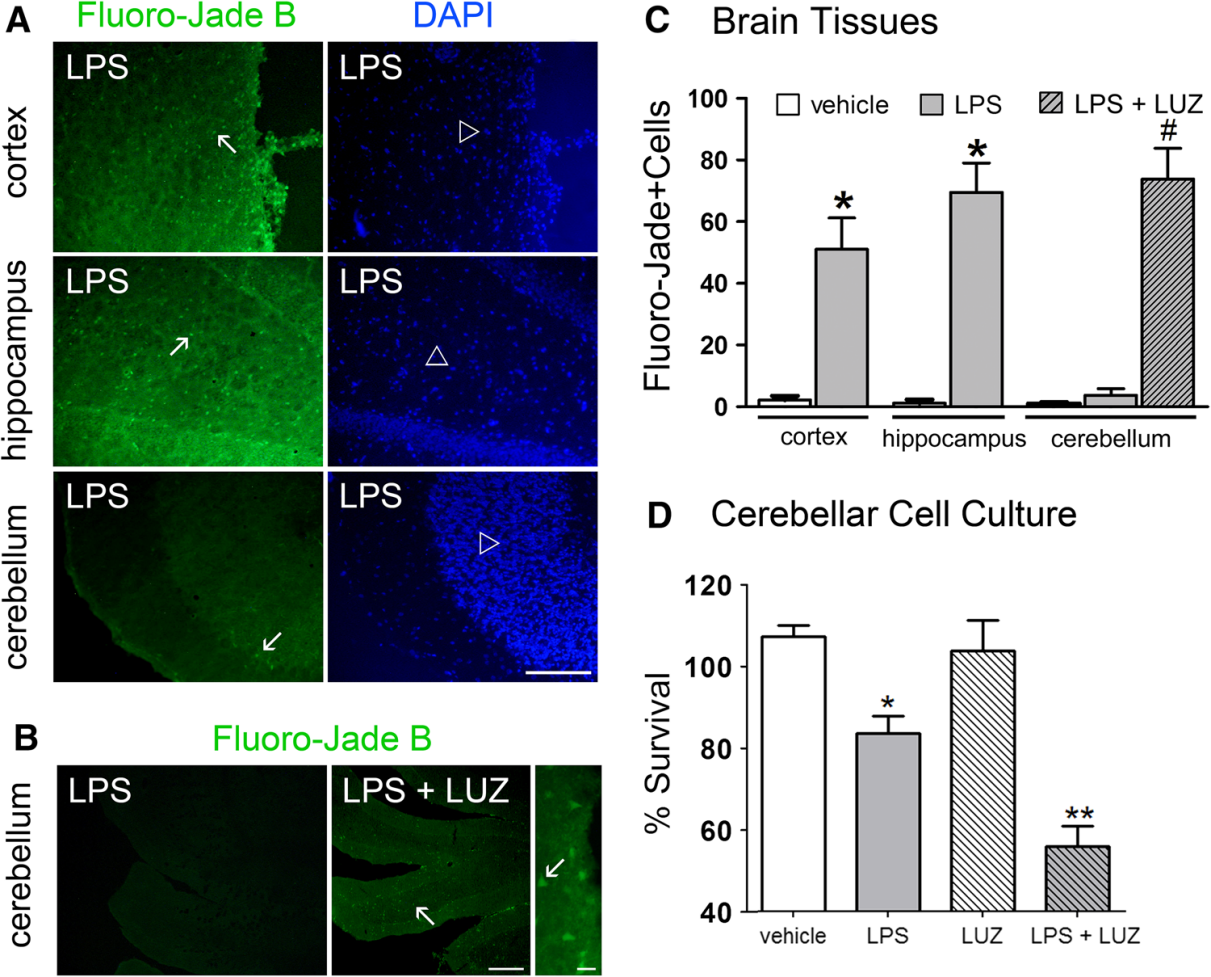
LPS-induced melatonin synthesis protected cerebellar neurons. **a** Neuronal degeneration was assessed through Fluoro-Jade B staining (*green*) in coronal slices of the cortex, hippocampus and cerebellum of LPS-treated rats (3 μg/5 μL, icv, ZT 6). Cell nuclei were stained with DAPI (*blue*). *Arrows* indicate dead cells, while *open arrowheads* indicate the nuclei of undamaged cells. *Scale bar* 100 μm. **b** Fluoro-jade B staining (*green*) of dead cells (*arrow*) in the cerebellum of LPS-treated animals (3 μg/5 μL, icv, ZT6) and animals injected with the melatonin receptor antagonist luzindole (10 μM, icv, ZT 5 and ZT 12) prior to LPS treatment. The nuclei of undamaged cells (*arrowhead*) were stained with DAPI (*blue*). *Scale bar* 20 μm. **c** Number of Fluoro-jade B positive cells in all three brain areas analyzed in the vehicle, LPS and LPS + luzindole (LPS + LUZ) groups (*n* = 4 animals per group). **d** Cell viability assay on cultured cerebellar cells challenged with LPS (30 ng/mL, 24 h) in the presence or absence of luzindole (100 nM, 24 h). Significant different from vehicle *(*p* < 0.01) or from luzindole **(*p* < 0.001). Significantly different from vehicle and LPS ^#^(*p* < 0.001)

Given that only the cerebellum exhibited local melatonin production and that melatonin receptors are expressed in cerebellar cells in mice (Imbesi et al. 2008), rats (Laudon et al. 1998), pigs (Williams et al. 1999) and humans (Al-Ghoul et al. 1998) we further investigated whether melatonin produced in the cerebellum could play a protective role mediated by melatonin receptors. Fluoro-Jade B staining indicated that LPS induces neuronal death in the cortex and hippocampus, but not in the cerebellum (Fig. 8a, c). When animals were treated with luzindole (50 μM, icv, ZT5 and ZT12), a competitive antagonist of melatonin receptors, the cerebellum became susceptible to LPS-induced neuronal death (Fig. 8b, c). This effect was further confirmed in cultured cerebellar cells in which the reduction in viability caused by LPS (100 ng/mL, 24 h) was potentiated in the presence of luzindole (100 nM, 24 h) (Fig. 8d). Therefore, our data suggest that the melatonin produced in the cerebellum acts through its membrane receptors to protect this area from LPS-induced neurotoxicity.

## Discussion

In this study, we show that inflammatory stimuli in the CNS activate the immune-pineal axis in a manner similar to systemic inflammation, leading to a switch between pineal and extra-pineal production of melatonin. In addition, locally produced melatonin protects the cerebellum from neuroinflammatory toxicity.

The most intriguing finding of this study is that LPS induced the synthesis of melatonin only in the cerebellum. In the cortex and hippocampus, LPS did not increase the concentration of melatonin above those found in tissues from control rats. After pinealectomy, the level of melatonin in both structures was highly reduced either in LPS-treated or control animals, indicating the importance of the pineal gland as a source of melatonin for the cortex and hippocampus. The content of melatonin in the cerebellum was significantly higher in pinealectomized animals, corroborating data obtained in rat liver (Venegas et al. 2012). However, the level of melatonin in the cortex and hippocampus was highly reduced, indicating that in our experimental conditions the cerebellum, but not the cortex and hippocampus, exhibits a local production of melatonin. This difference is unlikely to be due to the modulation of AA-NAT expression because AA-NAT-ir was increased by LPS (icv) treatment in cells stained with ED-1 both in the cortex and in the cerebellum, and in all the areas when cells were stained with IBA1. Therefore, the activation of the inflammatory response in the brain by LPS leads to an increase in AA-NAT-ir in all areas evaluated, as predicted by the immune-pineal axis hypothesis, although only the cerebellum produced melatonin.

Our analysis of AA-NAT expression in different cell types revealed that LPS significantly increased its expression primarily in cerebellar glial cells. Contrasting responses of different brain areas were also observed in a study of basal and LPS-induced expression of cytokines (Kipp et al. 2008), which also depends on the activation of glial cells. Different glial phenotypes following brain infection can lead to either detrimental or protective activity (Butovsky et al. 2001, 2005). This dual effect is strictly dependent on the area evaluated and drives the specific susceptibility of different cell types to injury conditions (Kim et al. 2000; Kipp et al. 2008; Espinosa-Oliva et al. 2011; Pintado et al. 2011). Although we cannot determine why, in our experimental conditions, the cerebellum, but not the hippocampus and cortex, is able to synthesize melatonin after pinealectomy we could show the mechanism of action. PDTC, which inhibits the binding of NF-κB dimers to κB regulating elements in the DNA, blocks the synthesis of melatonin by the cerebellum. It is well known that NF-κB can mediate both neurodegenerative and neuroprotective responses depending on the cell type and/or stimulus (O’Neill and Kaltschmidt 1997; Rivest 2009). The data reported here suggest that the induction of melatonin synthesis might comprise one of the downstream mechanisms by which NF-κB induced protective responses, as melatonin cytoprotective effects are well described (Galano et al. 2011, 2013; Pandi-Perumal et al. 2012).

The ability of LPS to induce neuronal death was less pronounced in the cerebellum when compared to the cortex or the hippocampus. However, when melatonin receptors were blocked, cerebellar neurons became susceptible to the neurotoxic effects of LPS, evidencing the role of melatonin G-protein coupled receptors in mediating its neuroprotective effect in an autocrine manner. Because the melatonin detected in the hippocampus is derived from the pineal gland, it is reasonable that the suppression of nocturnal melatonin surge by pinealectomy induces neuronal loss in the hippocampus, which is reversed by melatonin administration (De Butte and Pappas 2007). The fact that not all brain areas react similarly to LPS is not surprising because neurodegenerative disorders often occur in brain region-specific patterns, suggesting differences in the activity and reactivity of glial cells (Hald and Lotharius 2005; Williams et al. 2007). In this context, the ability to produce melatonin and to express melatonin receptors emerges as a relevant factor that might distinguish between protective and detrimental neuroinflammatory responses of the glial cells.

Traditionally, the neuroprotective effects of melatonin and its metabolites have been associated with antioxidant effects (Galano et al. 2011, 2013). A number of different mechanisms mediates this action, such as the direct interaction and scavenging of reactive species of oxygen and nitrogen or by genomic effects, leading to increase in the expression of antioxidant enzymes, or by preserving mitochondrial function (De Butte and Pappas 2007; Rennie et al. 2009; Cardinali et al. 2010; Fan and van Bel 2010; Wang 2009; Hashimoto et al. 2012; Srinivasan et al. 2011; Alonso-Alconada et al. 2012; Cary et al. 2012). Nevertheless, the involvement of melatonin G-protein coupled receptors MT_1_ and MT_2_ in mediating melatonin protective effects has also been previously described in studies of sepsis, apoptosis and brain injury (Fink et al. 2013; Husson et al. 2002; Radogna et al. 2007; Wang et al. 2013).

The expression of both MT_1_ and MT_2_ melatonin receptors has been demonstrated in mice (Imbesi et al. 2008), rat (Laudon et al. 1998), pig (Williams et al. 1999) and human (Al-Ghoul et al. 1998). Recently, using a transgenic mouse, the expression of MT_1_ melatonin receptors was confirmed in Purkinje cells and in the molecular and granular layers of the cerebellum (Adamah-Biassi et al. 2013), corroborating previous data using 2-[^125^I]-iodomelatonin assay in rats (Laudon et al. 1998). Moreover, cerebellar granule cells in culture also exhibit functional melatonin receptors (Huan et al. 2001). One of the first reported roles of melatonin receptors in the cerebellum refers to the maintenance of human equilibrium (Fraschini et al. 1999). Recently melatonin MT_2_ receptors were shown to stimulate the migration of rat cerebellar granule cells mediated by delayed rectifier outward potassium current (Liu et al. 2007a). Here, we show that melatonin acts as an autacoid in the cerebellum, interacting with its own receptors in the same area that it is synthesized. Blocking melatonin receptors with luzindole (icv) increased the neurotoxic effect of LPS. Although the intracellular pathways triggered by the activation of melatonin receptors in this condition still remain to be elucidated, the overall protective effect of melatonin usually involves its modulatory action on the innate immune response, reducing the synthesis of pro-inflammatory cytokines by lymphocytes (Lardone et al. 2010; Gupta and Haldar 2013), and enhancing phagocytosis by macrophages (Pires-Lapa et al. 2013).

In summary, here we show that the immune-pineal axis is activated following a neuroinflammatory response in the brain. The absence of pineal-derived melatonin may have further consequences, not only for the organization of the circadian system, but also for the susceptibility to neurodegenerative processes, especially because not all the brain areas are able to produce melatonin. In this regard, the ability to synthesize melatonin confers a special protection to cerebellar cells. The understanding of where, when and how melatonin is produced and acts in distinct brain areas is of clinical relevance since it provides valuable clues to guide the establishment of efficient protocols for the therapeutic use of this hormone in neuroinflammatory diseases.

## Electronic supplementary material

Below is the link to the electronic supplementary material.

Figure 1 The effect of pinealectomy on plasma concentration of melatonin at nighttime. The level of plasma melatonin in animals submitted (px) or not (naïve and sham) to pinealectomy determined by ELISA. In the pinealectomized and sham groups the quantification were realized 10 days after surgery. Daytime and Nighttime animals were killed at 12:00 and 24:00 hours, respectively. The data are expressed as the mean ± SEM of nine animals per group. *p < 0.05

Supplementary material 1 (TIFF 609 kb)
