# Supplementary figures and images for: Selective protection of the cerebellum against intracerebroventricular LPS is mediated by local melatonin synthesis
Source: Brain Struct Funct. 2013 Dec 22;220(2):827–40. doi: 10.1007/s00429-013-0686-4 (PMC4341011; doi:10.1007/s00429-013-0686-4)

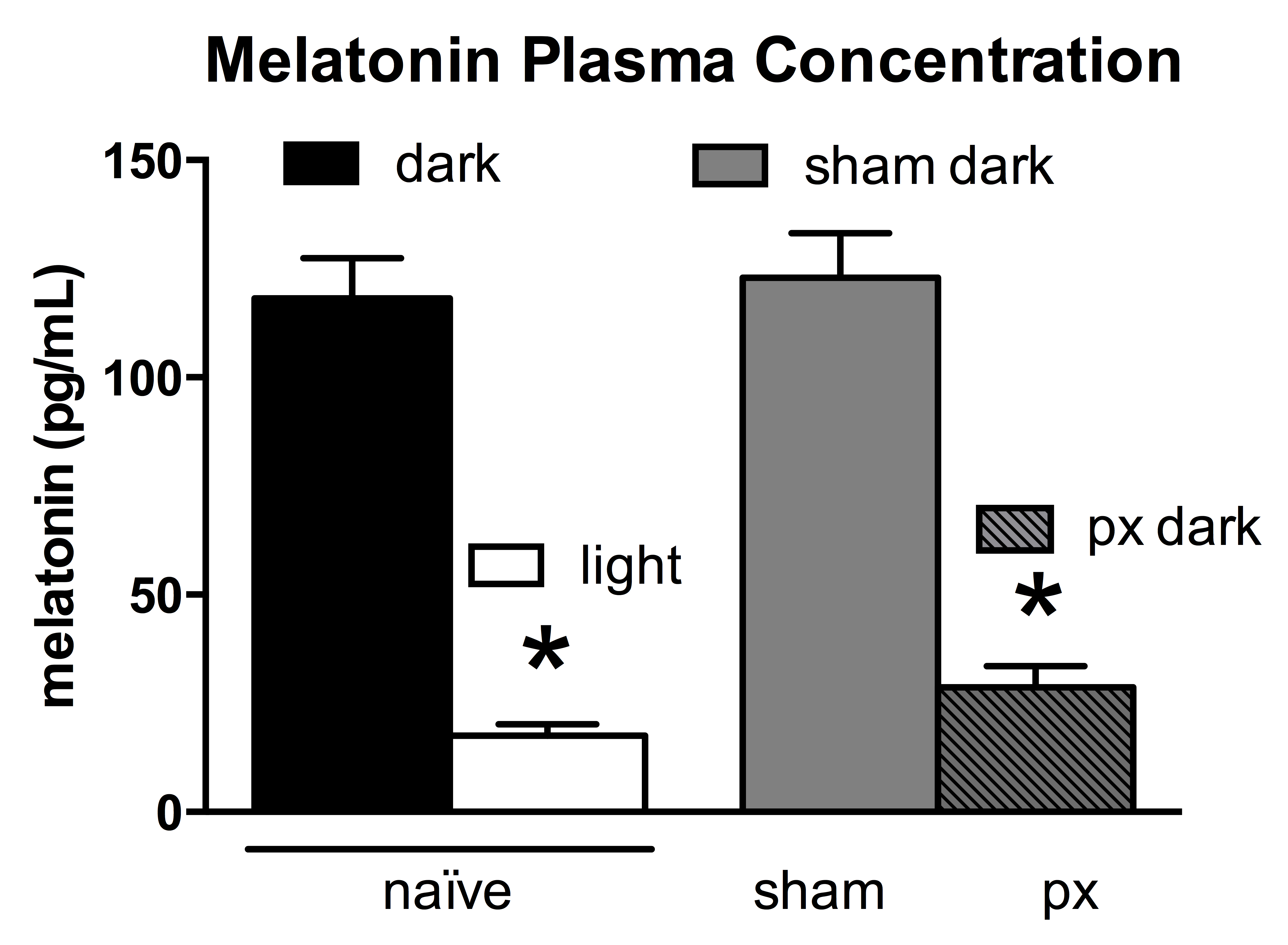

Supplement: Supplementary file 1 — Supplementary material 1 (TIFF 609 kb) [file 429_2013_686_MOESM1_ESM.tiff]
